# Acute postnatal inflammation alters adult microglial responses to LPS that are sex-, region- and timing of postnatal inflammation-dependent

**DOI:** 10.1186/s12974-024-03245-x

**Published:** 2024-10-10

**Authors:** Maria Nikodemova, Jose R. Oberto, Ethan L. Kaye, Mackenzie R. Berschel, Alysha L. Michaelson, Jyoti J. Watters, Gordon S. Mitchell

**Affiliations:** 1 Breathing Research and Therapeutic Center, Department of Physical Therapy and McKnight Brain Institute, University of Florida, Gainesville, FL 32610 USA; 2 Department of Comparative Biosciences, School of Veterinary Medicine, University of Wisconsin, Madison, WI 53706 USA

## Abstract

**Background:**

Adverse events in early life can have impact lasting into adulthood. We investigated the long-term effects of systemic inflammation during postnatal development on adult microglial responses to lipopolysaccharide (LPS) in two CNS regions (cortex, cervical spinal cord) in male and female rats.

**Methods:**

Inflammation was induced in Sprague-Dawley rats by LPS (1 mg/kg) administered intraperitoneally during postnatal development at P7, P12 or P18. As adults (12 weeks of age), the rats received a second LPS dose (1 mg/kg). Control rats received saline. Microglia were isolated 3 h post-LPS followed by gene expression analysis *via* qRT-PCR for pro-inflammatory (IL-6, iNOS, Ptgs2, C/EBPb, CD14, CXCL10), anti-inflammatory (CD68, Arg-1), and homeostatic genes (P2Y12, Tmemm119). CSF-1 and CX3CL1 mRNAs were analyzed in microglia-free homogenates.

**Results:**

Basal gene expression in adult microglia was largely unaffected by postnatal inflammation. Adult cortical microglial pro-inflammatory gene responses to LPS were either unchanged or attenuated in rats exposed to LPS during postnatal development. Ptgs2, C/EBPb, CXCL10 and Arg-1 were the most affected genes, with expression significantly downregulated vs. rats without postnatal LPS. Spinal microglia were affected most by LPS at P18, with mixed and sometimes opposing effects on proinflammatory genes in males vs. females. Overall, male cortical vs. spinal microglia were more affected by postnatal LPS. Females were affected in both cortex and spinal cord, but the effect was dependent on timing of postnatal LPS. Overall, inflammatory challenge at P18 had greater effect on adult microglia vs. challenge at P12 or P7.

**Conclusions:**

Long-lasting effects of postnatal inflammation on adult microglia depend on postnatal timing, CNS region and sex.

**Supplementary Information:**

The online version contains supplementary material available at 10.1186/s12974-024-03245-x.

## Introduction

Early life adverse events are associated with long-lasting health effects, including increased risk for neurodevelopmental, behavioral, and adult-onset neurodegenerative disorders [1–3]. Both humans and rodents are born with immature central nervous and immune systems that continue developing during the early postnatal period. Thus, infections or stress experienced during this critical developmental window may interfere with normal development and reprogram the immune response to challenges in adulthood [4–6]. Indeed, prenatal and neonatal infections are linked to schizophrenia, autism, ADHD and other behavioral disorders [3, 7].

Studies of postnatal inflammation induced by the TLR2/4 agonist lipopolysaccharide (LPS) report increased risk of seizure [8] and impaired plasticity of cervical spinal motor neurons [9–11] in adult rats. In rodents, neonatal (P3-P5) LPS exposure causes robust activation of microglia, the resident CNS immune cells. In association, neonatal LPS causes inflammatory injury of dopaminergic neurons [12], enhanced neurogenesis, and decreased developmental apoptosis in the hippocampus and subventricular zone, with associated learning deficits [13, 14]. Brain IL1β is persistently elevated after neonatal LPS, at least until post-natal day 70 [12, 14]. Finally, neonatal (P4) infections in rodent models are associated with exacerbated memory impairment after immune challenge in adulthood due to abnormal IL1β and BDNF regulation [15–17]. Thus, neonatal inflammation causes multiple neurodevelopmental disorders that persist into adulthood.

Normal postnatal CNS development is characterized by synaptogenesis, synaptic pruning, neuronal apoptosis and myelination, and microglia play a critical role in these processes [18–20]. During development, microglia undergo morphological and phenotypic transformation, reflecting their evolving roles throughout development. A recent study in mice revealed a stepwise developmental program in microglial gene expression during embryonic and early postnatal life between E10.5-P9 [21]. However, this study did not evaluate later postnatal stages. There are significant differences in murine microglial gene expression between P3 and P21; whereas pro-inflammatory mediators, such as TNFα and iNOS, are elevated at postnatal day 3, CD11b, TLR4, FcRγ and P2X  7 are more highly expressed in P21 microglia [22, 23].

In rodent models, microglia proliferate and colonize the CNS during the first 2 postnatal weeks. During this period, microglial density is twofold higher vs. adult brains [24, 25]. Microglial numbers begin to decline in the third postnatal week, reaching adult numbers by week 6 [24]. Although microglial developmental trajectories in humans are less understood since they require postmortem tissues, it was shown that microglia first appear in ventricular, intermediate and marginal zones at 4.5 gestational weeks; they colonize other CNS regions until 24 weeks of gestation [26]. Microglial density is highest in the early fetal and neonatal/infancy periods, followed by increased microglial cell death [27]. High microglial density coincides with neurogenesis and synaptic pruning, consistent with a role for microglia in these processes [27], similar to rodents. In healthy adult brains, microglia contribute to neural homeostasis and immune function [28, 29]. With CNS pathology and/or trauma, microglia exhibit both beneficial and detrimental activities. Considerable evidence underscores the importance of understanding how microglial activities are shaped by experiences that occur during development vs. adulthood.

Most studies of inflammation in early development focus on challenges during the fetal and neonatal period. Considerably less is known about the long-term effects of inflammation in later postnatal developmental stages. Here, we investigated the impact of acute systemic inflammation triggered by intraperitoneal LPS administration to P7, P12, or P18 rats on microglial responses to the same challenge as adults. These postnatal time points coincide with key developmental stages of the CNS in general, and microglia in specific. In P7 rats, neurogenesis, axonal growth and microglial proliferation are well underway, followed by robust synaptogenesis, synaptic pruning, and programmed cell death at P12 and P18 [30]. At P12, microglial numbers peak, whereas microglia have reduced proliferative capacity and their numbers decline due to apoptosis at P18 [24, 31]. Thus, we hypothesized that inflammatory insults at these distinct developmental stages would have differential effects on adult microglia.

Since microglia exhibit regional differences during development and in adults [30, 32], we further hypothesized that their vulnerability to inflammatory insult is CNS region-specific. Thus, we evaluated microglia in the cortex and cervical spinal cord, since the latter is critical for essential forms of respiratory motor plasticity yet is seldom considered in studies concerning early life inflammation effects on adult function. Indeed, neonatal inflammation has profound effects on spinal respiratory motor plasticity [9, 11], highlighting the need for greater understanding of how inflammation in early life impacts adult spinal microglia. Finally, we determined if long-lasting effects of postnatal LPS on adult microglia are sex dependent.

## Methods

### Animals

All experiments were approved by the University of Florida Institutional Animal Care and Use Committee. Pregnant Sprague-Dawley rats were purchased from Envigo (IN, USA). Rats were housed at AAALC-accredited animal facility under standard conditions with a 12-hour light/dark cycle and *ad libitum* food and water.

### Experimental design

**Fig. 1:**
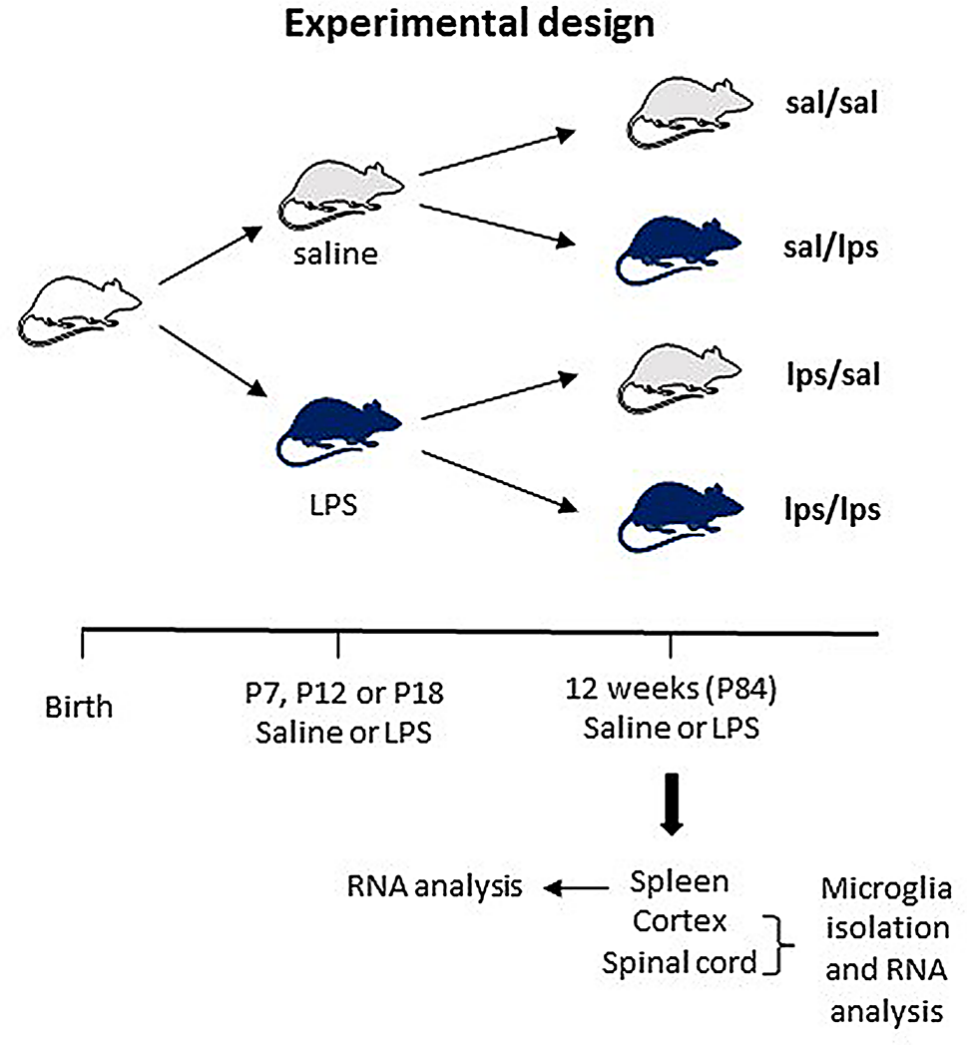
**Experimental design.** Male and female pups in each litter were randomly divided into two subgroups receiving either i.p. saline or LPS (1 mg/kg) at P7, P12 or P18. At 12 weeks of age (P84), animals in each subgroup received either saline or LPS (1 mg/kg). Three hours post-injection animals were perfused followed by spleen, cortex and cervical spinal cord harvest. Fresh CNS tissues were used for microglial isolation and gene expression analysis by qRT-PCR. The experimental group name depicts postnatal/P84 treatment

Experimental design and rat groups are depicted in Fig. 1. At P7, P12 or P18, pups in each litter were randomly assigned to subgroups receiving either saline or LPS (O111:B4 serotype; Sigma) at 1 mg/kg administered *via* i.p. injection. For each time point, rats from 2 to 3 litters were included to balance inter-litter differences. Rats were then grown to 12 weeks of age when rats from each subgroup were randomly assigned to receive a second challenge of either saline or LPS (1 mg/kg, *i.p*.). Thus, rats from each litter were distributed in a way that they received all experimental treatments, thereby minimizing inter-litter variations. LPS was administered between 9 and 10 am in postnatal rats and 8.30 am -12pm in adult rats to avoid potential circadian or other diurnal effects on the inflammatory response. We chose a relatively high LPS dose to induce moderate-to-severe inflammation, while avoiding life-threatening sepsis. At this dose, there was no mortality (100% survival), and we did not observe major behavioral or body changes (posture, swelling) after LPS in any group. Table 1 depicts rat numbers in each experimental group. Adult rats were deeply anesthetized with isoflurane 3 h after saline or LPS injections, and the rats were then intracardially perfused with ice-cold PBS followed by dissection of the spleen, the entire cortex, and the cervical spinal cord. CNS tissues were immediately subjected to microglial isolation. Spleen tissues were frozen and stored at -80 °C until further use.

**Table 1:** Group size (n) across experimental groups. Groups are named based on their postnatal/adult treatment. Sal, saline; LPS, lipopolysaccharide; P7 postnatal day 7; P12, postnatal day 12; P18, postnatal day 18

Treatment	Males	Females
P7/adult		
sal/lps	7	7
lps/sal	6	8
sal/lps	6	7
lps/lps	8	9
**P12/adult**		
sal/lps	9	5
lps/sal	8	4
sal/lps	9	5
lps/lps	8	5
**P18/adult**		
sal/lps	9	8
lps/sal	5	12
sal/lps	10	7
lps/lps	6	13

### Microglia isolation

Microglia were isolated from fresh tissues using an immunomagnetic cell separation method described in detail previously [33]. All reagents were obtained from Miltenyi Biotech, Germany. Tissues were mechanically and enzymatically dissociated into single cell suspensions using a papain Neural Tissue Dissociation Kit. After myelin removal by centrifugation in Debris Removal Solution, cells were incubated with CD11b magnetic beads followed by separation in a magnetic field using MS columns. Both CD11b^+^ (microglia) and CD11b^−^ (non-microglia) fractions were collected. This method consistently yields > 96% microglial cell purity [33].

After tissue dissociation, a small part of sample was analyzed by flow cytometry to determine the proportion of CD45^high^/CD45^low^ among CD11b cells (Supplementary Fig. 1). Around 98% of CD11b cells were CD45^low^ (microglia) and around 2% of cells were CD45^high^ (presumably macrophages). This proportion did not change after LPS treatment or by prior LPS exposure suggesting that treatments did not induce macrophage infiltration.

### RNA extraction and qRT- PCR

Total RNA was extracted from cells using TRIZOL reagent (Invitrogen) following the manufacturer’s protocol. Trans-sectional spleen slices were ultrasonically homogenized in 1 ml TRIZOL reagent followed by total RNA extraction. Extracted RNA (250 ng) was used for cDNA synthesis using iScript Advanced cDNA Synthesis kit (BioRad). All primers for quantitative PCR were obtained from BioRad (Table 2) together with SsoAdvanced Universal Real-Time PCR Supermix. After normalization to the reference gene *hprt1*, gene expression was analyzed *via* the ΔΔCt method. Ct values of *hprt1* for each experimental group are in Supplementary Fig. 2.

**Table 2:** List of genes analyzed by qRT-PCR

Gene	Description	Function	BioRad primer
P2Y12	Purinergic membrane receptor	homeostatic microglia	qRnoCED0002978
Tmem119	Transmembrane protein 119	homeostatic microglia	qRnoCED0010608
CD68	Transmembrane glycoprotein (scavenger receptor family)	Phagocytosis Anti-inflammation	qRnoCED0005201
CD14	Membrane receptor; co-receptor for TLR4	Inflammation	qRnoCED0008863
CXCL10	cytokine	Inflammation	qRnoCED0009075
C/EBPb	CCAAT-enhancer-binding protein; transcription factor	Inflammation	qRnoCED0006139
IL-6	Interleukin 6; cytokine	Inflammation	qRnoCID0053166
Ptgs2	Prostaglandin-endoperoxide synthase 2; also known as COX2	Inflammation	qRnoCED0002406
iNOS	Inducible nitric oxide synthetase	Inflammation	qRnoCED0020417
Arg-1	Arginase 1, competing with iNOS for substrate	Anti-inflammation	qRnoCID0006520
Hprt1	Hypoxanthine phosphoribosyltransferase 1	Reference gene	qRnoCED0057020
CSF-1	Colony stimulatory factor 1	Growth factor	qRnoCID0004474
CX3CL1	C-X3_C motif chemokine ligand 1	Chemokine	qRnoCED0007474

### Statistical analysis

Data were analyzed using SigmaPlot (descriptive statistics, ANOVAs) and SAS (regression analysis), and R software, vegan package (Bray-Curtis dissimilarity index and PERMANOVA). Data are expressed as mean ± 1 standard error of mean (SEM). Individual gene expression was analyzed by one-way ANOVA followed by *post hoc* Tukey tests. Two-way ANOVAs (sex-by treatment) were used to assess sexual dimorphism in LPS-evoked adult microglial gene expression following postnatal LPS exposure. Differences were considered significant if *p* < 0.05.

Overall differences in gene expression in response to LPS between rats with and without postnatal LPS treatment was analyzed by Bray-Curtis (BC) dissimilarity index, a statistical tool quantifying the dissimilarity between groups based on the group composition commonly used in ecological and microbiological studies. The Bray-Curtis dissimilarity analysis calculates within- and inter- group distances of individual samples (where sample composition is determined by proportion of individual gene RNA), PERMANOVA tests then identify significant differences between groups, considering both within and inter-group variability. The BC dissimilarity index is bounded between 0 and 1; 0 indicates two groups are identical, and 1 means they are completely different. For detailed description of “dissimilarity index,” see Supplementary Information and Supplemental Fig. 3.

## Results

### Early-life immune challenge does not affect adult body weight

**Fig. 2:**
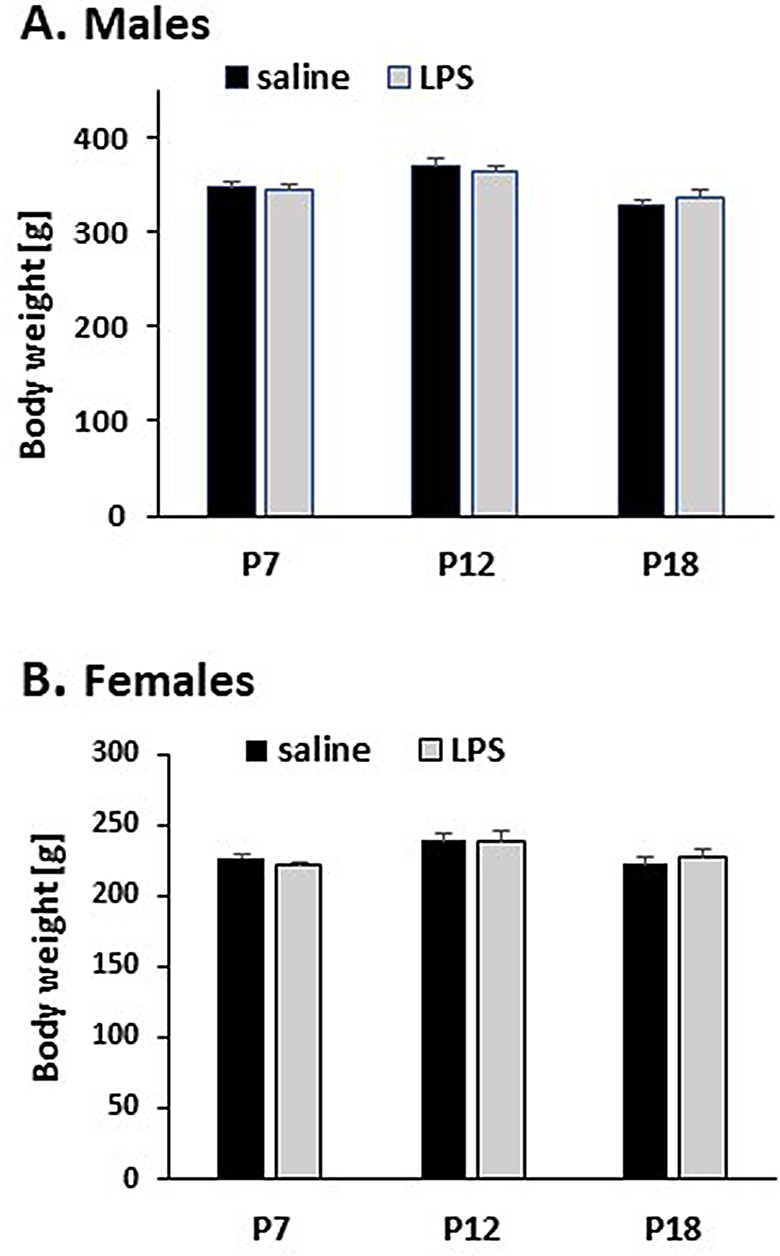
**The effect of postnatal LPS challenge on adult body weight.** Body weight was recorded at P84 before adult challenge with saline or LPS. The legend (saline, LPS) indicates postnatal treatment. We did not observe any significant changes in body weight in males (**A**) or females (**B**) receiving LPS postnatally compared to rats receiving only saline

Inflammation induced by LPS during postnatal development did not affect body weight (BW) at 12 weeks of age (Fig. 2). Body weights were recorded before the second LPS or saline challenge. The average BWs of males receiving saline or LPS during postnatal development (P7, P12, P18) were 349 g and 351 g, whereas the average BWs of females were 228 g and 228 g, respectively.

### Adult microglial responses to LPS differ by CNS region and sex

**Fig. 3:**
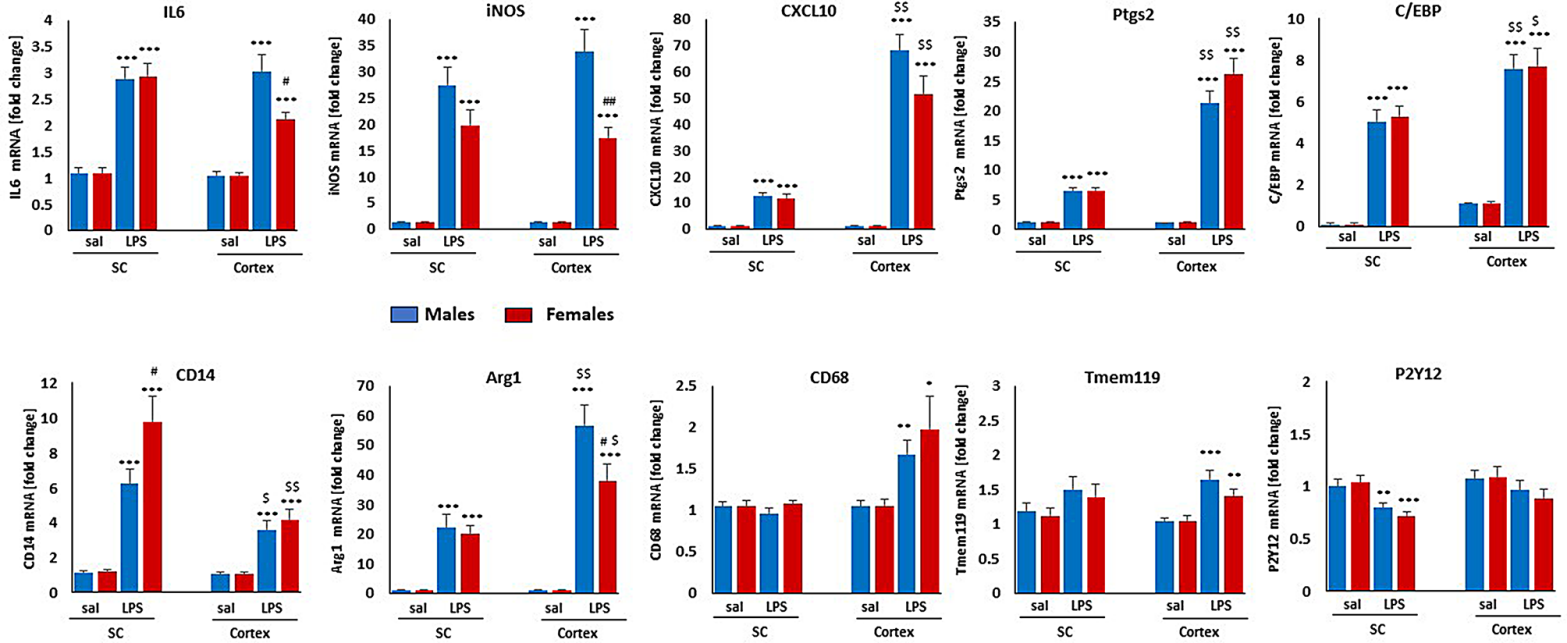
**Adult microglial responses to LPS.** Rats receiving only saline during postnatal development were used as controls to determine adult microglia response to LPS (1 mg/kg) in cortex or cervical spinal cord. Gene expression was analyzed 3 h post LPS administration. Blue: males, red: females. Statistical significance is illustrated as follows: * LPS vs. saline; # males vs. females; $ cortex vs. spinal cord. One symbol = *p* < 0.05, two symbols *p* < 0.01, three symbols *p* < 0.001

We first evaluated adult microglial responses to systemic LPS in adult rats that had received saline during postnatal development (controls) 3 h post-LPS or saline administration, including genes associated with inflammation, phagocytosis and microglial homeostasis (Table 2). Three hours post-LPS treatment, IL6, iNOS, CXCL10, Ptgs2, C/EBPb, CD14 and Arg1 were significantly upregulated in adult male and female microglia in both cortex, cervical spinal cord, although the magnitudes of individual gene responses differed (Fig. 3). Cortical microglia from both sexes had significantly higher levels of CXCL10, Ptgs2, C/EBPb and Arg1 after LPS *versus* spinal microglia; the CD14 response was significantly lower in cortical microglia. Increased CD68 and Tmem119 expression post-LPS was detected only in cortical microglia. On the other hand, P2Y12 was downregulated after LPS exclusively in spinal microglia. We detected only few sex-dependent differences in response to LPS; cortical male microglia had significantly higher expression of IL6 (by 40%), iNOS (by 95%) and Arg1 (by 49%) then females. In spinal microglia, the only sex difference identified was in CD14, which was higher in female (by 56%) versus male microglia after LPS. We did not detect changes in TNFα, CCL2 or IL1β mRNA 3 h post-LPS (data not shown).

### Early-life inflammation has little effect on basal gene expression in adult microglia

To determine if postnatal inflammation had long-lasting effects on adult microglia, we evaluated basal gene expression in 12-week-old rats exposed to LPS at P7, P12 or P18. In microglia from cortex and spinal cord, a single LPS challenge during postnatal development had minimal effects on basal expression of any gene evaluated here (Table 3). Spinal microglial IL6 expression was downregulated in females exposed to LPS at P7, and in males exposed to LPS at P18. In cortical microglia, there were no significant changes in gene expression in females; in male cortical microglia, CXCL10 mRNA was elevated in rats receiving LPS at P7, and CD14 was downregulated in adult rats exposed to LPS at P18.

**Table 3:** Multivariate linear regression analysis of microglial basal gene expression. Data show intercept (control animal receiving saline in postnatal period), β (SE) and p-values for sex and time of postnatal LPS exposure for each gene. Ns, not significant. Male basal gene expression (sal/sal) was set to 1 for each gene and gene expression in females was calculated relative to males

Cortex	IL6	iNOS	Arg-1	Ptgs2	C/EBP	Cxcl10	CD14	CD68	Tmem119	P2Y12
intercept	1.01 (0.08)	1.15 (0.11)	1.26 (0.19)	1.03 (0.08)	1.06 (0.08)	1.37 (0.48)	1.07 (0.07)	1.04 (0.06)	1.06 (0.06)	1.07 (0.08)
Sex (F)	0.05 (0.10) ns	-0.00 (0.13) ns	-0.28 (0.22) ns	0.04 (0.10) ns	-0.00 (0.09) ns	-0.70 (0.58) ns	-0.01 (0.08) ns	0.00 (0.08) ns	-0.05 (0.07) ns	0.00 (0.10) ns
P7	-0.18 (0.13) ns	-0.36 (0.18) ns	0.27 (0.31) ns	0.00 (0.13) ns	-0.22 (0.13) ns	2.07 (0.80) *p* < 0.0120	-0.16 (0.11) ns	0.04 (0.10) ns	0.01 (0.10) ns	-0.03 (0.14) ns
P12	0.10 (0.14) ns	-0.16 (0.19) ns	0.70 (0.31) *P* < 0.0303	0.03 (0.14) ns	0.23 (0.14) ns	0.15 (0.83) ns	0.08 (0.11) ns	0.22 (0.11) *P* < 0.0488	0.12 (0.10) ns	0.05 (0.146 ns
P18	-0.33 (0.14) *P* < 0.0218	-0.18 (0.18) ns	-0.00 (0.32) ns	-0.00 (0.14) ns	0.02 (0.13) ns	0.13 (0.82) ns	-0.19 (0.11) ns	-0.05 (0.11) ns	-0.07 (0.10) ns	0.02 (0.14) ns
Cervical Spinal Cord	**IL6**	**iNOS**	**Arg-1**	**Ptgs2**	**C/EBP**	**Cxcl10**	**CD14**	**CD68**	**Tmem119**	**P2Y12**
intercept	1.06 (0.08)	1.22 (0.13)	1.08 (0.24)	1.14 (0.09)	1.18 (0.13)	1.03 (0.07)	1.11 (0.10)	1.02 (0.06)	1.17 (0.12)	1.00 (0.06)
Sex (F)	0.04 (0.09) ns	-0.05 (0.16) ns	0.12 (0.29) ns	-0.06 (0.11) ns	-0.03 (0.16) ns	0.06 (0.08) ns	-0.01 (0.12) ns	0.02 (0.08) ns	-0.04 (0.14) ns	-0.01 (0.07) ns
P7	-0.36 (0.14) *p* < 0.0122	-0.12 (0.23) ns	-0.04 (0.41) ns	-0.12 (0.15) ns	-0.36 (0.23) ns	0.10 (0.12) ns	-0.15 (0.18) ns	0.18 (0.11) ns	0.06 (0.20) ns	-0.06 (0.10) ns
P12	-0.19 (0.14) ns	-0.47 (0.23) *P* < 0.0506	0.01 (0.43) ns	-0.32 (0.16) *p* < 0.0518	-0.22 (0.23) ns	0.03 (0.12) ns	-0.03 (0.18) ns	-0.02 (0.12) ns	-0.10 (0.21) ns	-0.02 (0.11) ns
P18	-0.22 (0.13) ns	-0.41 (0.21) ns	0.50 (0.40) ns	-0.14 (0.1) ns	0.08 (0.21) ns	0.09 (0.11) ns	0.04 (0.17) ns	0.03 (0.11) ns	0.18 (0.19) ns	0.01 (0.10) ns

### LPS-evoked gene expression differs with early-life exposure timing, CNS region and sex

**Fig. 4:**
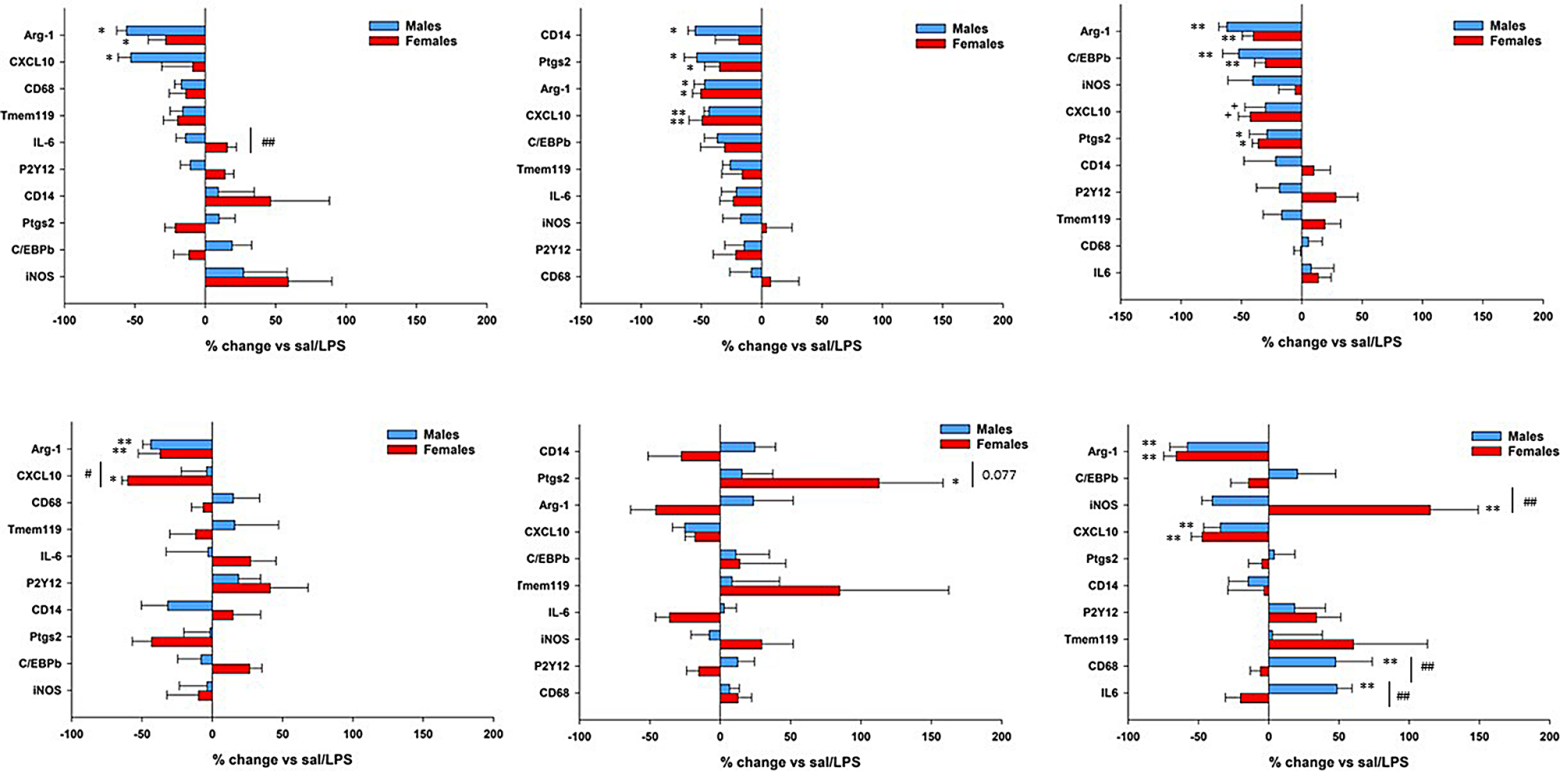
**The effect of postnatal inflammation on adult microglial responses to LPS.** Animals received first dose of LPS (1 mg/kg) either at P7, P12 or P18. Second LPS (1 mg/kg) was administered at P84 (12 weeks of age). Adult males **(blue)** and females **(red)** microglial responses in cortex and cervical spinal cord were compared to control animal receiving LPS only at P84. To reveal a potential pattern in altered microglial responses, genes are organized from most negative to most positive in male cortical microglia for each time point (P7, P12, P18). The same gene order was then used for spinal cord. *p < 0.05, **p < 0.01, ***p < 0.001 vs. sal/LPS; ^#^*p* < 0.05, ^##^*p* < 0.01 males vs. females

Postnatal LPS effects on adult microglial inflammatory responses were dependent on CNS region, sex and the timing of postnatal LPS delivery (Fig. 4). Cortical microglial responses were attenuated in males exposed to LPS at P12 and P18 vs. controls (i.e., saline at P12 or P18; Fig. 4). The most affected genes were CXCL10, C/EBPb, CD14 and Ptgs2. P7 LPS treatment had lesser effects on male adult microglia; only the CXCL10 response was significantly attenuated. In females, cortical microglial inflammatory responses were largely unaffected by postnatal LPS at P7; in contrast, expression of several pro-inflammatory genes was attenuated by P12 or P18 LPS pretreatment (Fig. 4). The only significant difference between male vs. female cortical microglia was IL6 in P7 treated rats, where male and female responses were in opposite directions. Although neither change reached statistical significance when compared with rats that had not received postnatal LPS (sal/LPS), the difference between them was significant.

We observed greater variability in spinal microglial responses (i.e., the SEM was larger for many genes, lessening the probability of detecting statistically significant changes). Adult spinal microglial pro-inflammatory responses were largely unaffected in rats that had received postnatal LPS at P7 or P12; two exceptions were a downregulation of CXCL10 in P7 treated females, and upregulation of Ptgs2 in P12 treated females, but not males. LPS at P18 had largest effects on adult spinal microglia, with several sex-dependent differences. iNOS was upregulated in females but not males, and CD68 and IL6 was upregulated in males but not females. Further, the CXCL10 response was significantly attenuated in both sexes (Fig. 4).

Responses of homeostatic microglial genes P2Y12 and Tmem119 to LPS as adults were unaffected by postnatal LPS in cortex or spinal cord. Similarly, adult LPS responses in CD68 (gene associated with phagocytosis) were unaffected by postnatal LPS, except in male spinal microglia, where it was significantly attenuated in P18 treated rats (Fig. 4). Regardless of timing, postnatal LPS exposure significantly blunted the Arg-1 response to adult LPS by up to 80% in cortical microglia (Fig. 4). In spinal microglia, only P7 and P18 exposure attenuated adult Arg-1 responses to LPS.

To further analyze differences in adult microglial responsiveness to LPS with and without postnatal LPS challenge, we calculated the Bray-Curtis dissimilarity (BC) index that reflects differences in expression of all 10 genes, followed by PERMANOVA test to determine statistical significance of group differences. The advantage of this analysis is that it encompasses differences in global gene expression (10 evaluated genes), even if changes are not statistically significant for individual genes. It is plausible that an accumulation of small (difficult to detect) differences across multiple genes could lead to biologically meaningful effects. To demonstrate suitability of the BC index for analyzing group differences, we performed a sensitivity analysis (Supplemental Fig. 3) demonstrating we can detect expected differences among rats receiving saline vs. LPS. Adult LPS-evoked microglial gene expression was significantly different from rats receiving saline, regardless of postnatal treatment (sal/sal vs. sal/LPS; LPS/sal vs. LPS/LPS) in both CNS regions and in both sexes (Table 4). There were no significant differences in adult basal gene expression with (LPS/sal) or without (sal/sal) postnatal LPS. However, there were significant differences in microglial LPS responses between rats receiving saline (sal/LPS) or LPS (LPS/LPS) postnatally. In males, cortical microglia were more sensitive to postnatal LPS than spinal microglia, regardless of post-natal timing. Although the dissimilarity index is comparable between spinal and cortical microglia, larger within group variability in spinal cord undermined the ability to detect significant differences. In females, cortical microglia appeared less affected than males overall; only LPS at P18 significantly affected adult responses to LPS; a similar trend at P12 was marginally significant, whereas no significant effects were detected in P7 treated rats. While male spinal microglia were not significantly affected by postnatal LPS, female spinal microglia were vulnerable to postnatal LPS at P7 and P18.

**Table 4:** Bray-Curtis dissimilarity index and Permutational MANOVA analysis for group differences. Data were analyzed for group difference in R, using vegan packages

	sal/sal vs. sal/LPS	*p*-value	sal/sal vs. LPS/sal	*p*-value	LPS/sal vs. LPS/LPS	*p*-value	sal/LPS vs. LPS/LPS	*p*-value
Cortex								
P7 males	0.577	**0.001**	0.258	ns	0.576	**0.001**	0.296	**0.003**
P12 males	0.607	**0.001**	0.260	ns	0.547	**0.001**	0.331	**0.001**
P18 males	0.577	**0.001**	0.211	ns	0.599	**0.012**	0.388	**0.004**
P7 females	0.565	**0.001**	0.230	ns	0.576	**0.001**	0.281	ns
P12 females	0.612	**0.008**	0.277	ns	0.599	**0.007**	0.296	0.059
P18 females	0.581	**0.001**	0.244	ns	0.540	**0.001**	0.318	**0.025**
**Spinal cord**								
P7 males	0.550	**0.001**	0.239	ns	0.533	**0.001**	0.270	ns
P12 males	0.530	**0.001**	0.253	ns	0.551	**0.001**	0.336	ns
P18 males	0.549	**0.001**	0.275	ns	0.513	**0.002**	0.341	ns
P7 females	0.519	**0.001**	0.272	ns	0.553	**0.001**	0.311	**0.045**
P12 females	0.548	**0.009**	0.272	ns	0.569	**0.002**	0.305	ns
P18 females	0.504	**0.001**	0.303	ns	0.501	**0.001**	0.321	**0.027**

### Attenuated LPS-induced CSF-1 gene expression in non-microglial cell fraction

**Fig. 5:**
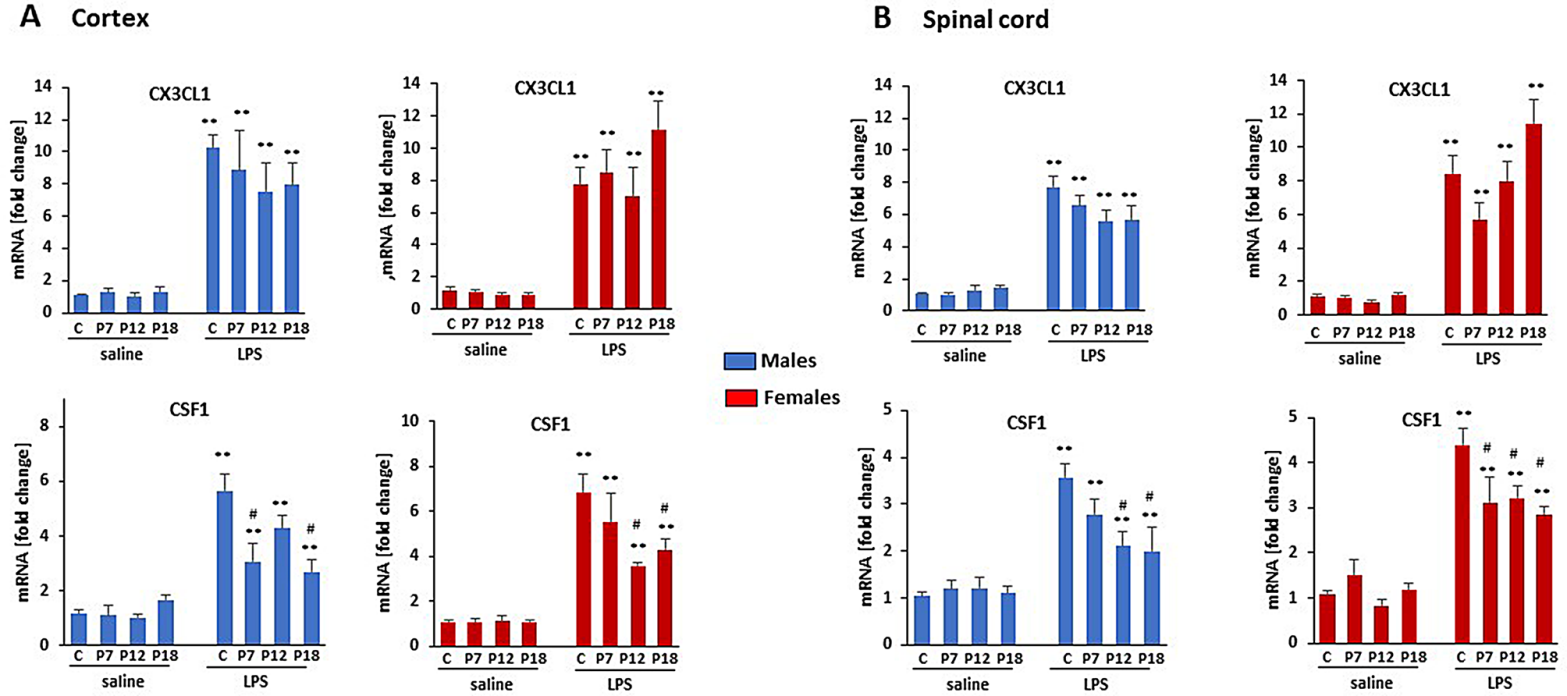
**The effect of postnatal inflammation on CX3CL1 and CSF1.** The expression of CX3CL1 and CSF1 was assessed in microglial-free tissue homogenates in cortex (**A**) and cervical spinal cord (**B.**) There was no difference in basal expression of these genes (saline) in adult rat with and without postnatal LPS exposure. P7, P12, P18 indicates when LPS was administered. “C” indicates that animals received only saline postnatally. LPS at 12 weeks of age induced significant upregulation on CX3CL1 and CSF1 in males **(blue**) and females (**red**) in both CNS regions. CX3CL1 and CSF1 were significantly upregulated by LPS both in cortex and spinal cord in animals receiving saline (C) or LPS (P7, P12, P18) postnatally. **p < 0.01 vs. saline; ^#^*p* < 0.05 vs. LPS in “C” animals

In the CD11b negative cell fractions (neurons, astrocytes, oligodendrocytes, endothelial cells, and others) from cortex and cervical spinal cord, we analyzed CX3CL1 (fractalkine) and CSF-1 (colony stimulating factor 1) expression since they are both important regulators of microglial activities (Fig. 5). CX3CL1 is expressed primarily by neurons whereas CSF-1 is produced by neurons and astrocytes. LPS significantly upregulated both genes in adult cortical and spinal CD11b negative cell fractions; however, postnatal LPS had no effect on basal or LPS-evoked CX3CL1 expression. On the other hand, the LPS-evoked increase in adult CSF-1 was attenuated by postnatal LPS in both regions, although this effect did not reach statistical significance in cortex from males treated with LPS at P12 (*p* = 0.243), females treated with LPS at P7 (*p* = 0.389), or spinal fractions from males treated with LPS at P7 (*p* = 0.169).

### LPS treatment at P18 significantly affects adult spleen response to LPS

**Fig. 6:**
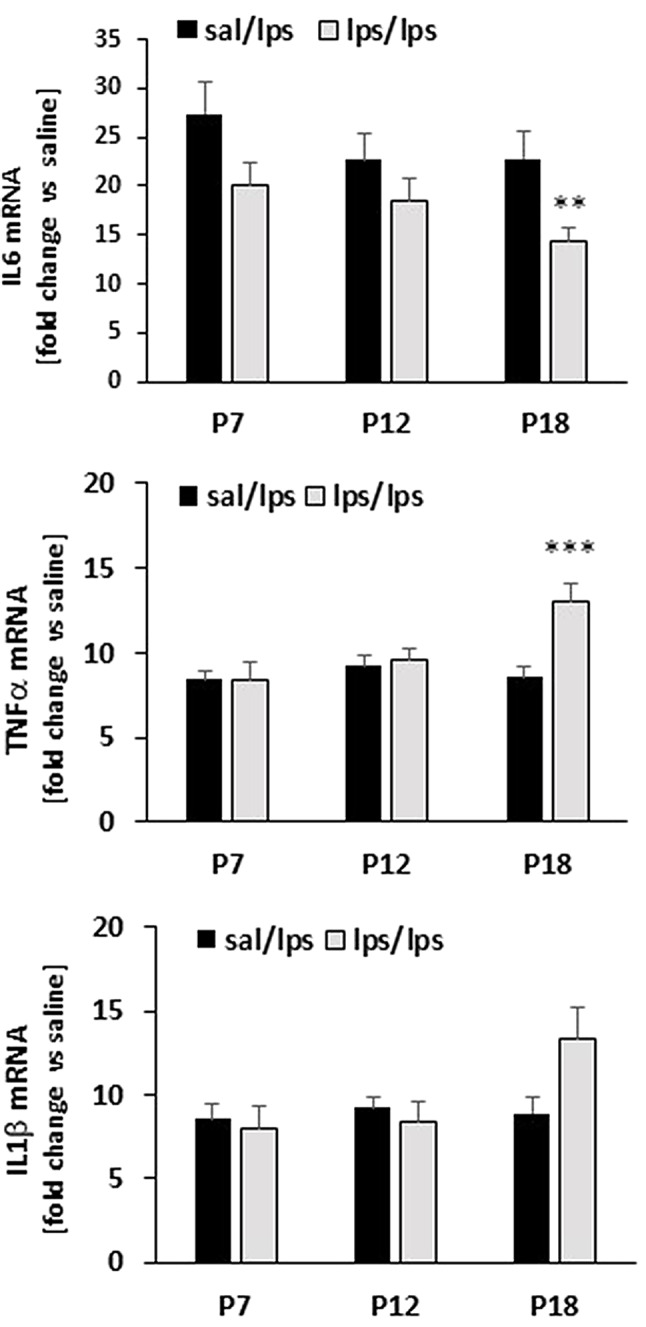
**The effect of postnatal inflammation on adult spleen responses to LPS. **Animals were exposed LPS or saline at P7, P12 or P18. At the age of 12 weeks, animals received either saline or LPS; gene expression was evaluated 3 h post LPS administration. Male and female data are pooled together. LPS induced significant upregulation of inflammatory cytokines in all treatment group. Statistical significance is marked only for sal/lps vs. lps/lps groups. **p < 0.01, ***p < 0.001 vs. sal/LPS

Because neuroinflammation was induced secondarily to systemic inflammation elicited via intraperitoneal LPS, we evaluated whether the peripheral LPS immune response in adults was affected by postnatal LPS challenge. We did not observe any differences in gene expression for IL6, TNFα or IL1β in basal (not shown) or LPS-evoked response of spleens from rats with P7 or P12 LPS challenge (Fig. 6). However, in adult spleens from rats exposed to LPS at P18, LPS-evoked IL6 mRNA levels were attenuated and TNFα mRNA levels exaggerated. We observed an apparent increase in LPS-evoked IL1β gene expression in spleens from rats treated with LPS at P18, but this change did not reach statistical significance (*p* = 0.136).

## Discussion

Microglia contribute to important biological functions in the developing and adult CNS including neurogenesis, myelination, synaptic remodeling, synaptic pruning and synaptic plasticity [20, 29, 34]. Microglial reactivity is observed in virtually all CNS pathologies or injuries, exerting both harmful and beneficial effects depending on their microenvironment and the specific pathology [29, 35]. The immune system, including microglia, is immature at birth and continues to develop for up to 7 weeks of age [36]. This period is a time of increased vulnerability during which adverse events can significantly impact development, potentially reprogramming microglial immune responses [36, 37]. Since CNS and immune system development continues during postnatal life, physiological challenge during development, such as acute inflammation, could differentially shape adult microglial responses, depending on the specific timing of the developmental challenge. Increasing evidence suggests that CNS disorders including Alzheimer’s disease, schizophrenia, attention deficit hyperactivity disorder, memory deficits, and autism may have developmental origins, encapsulated in the “two-hit” hypothesis [1, 2, 6, 38, 39]. In this hypothesis, the first hit in early life, such as infection, traumatic brain injury or stress, determines responses to a second hit experienced later in life. Most studies to date focus on long-term effects of maternal, fetal or neonatal immune challenges. Far less is known concerning long-term effects of infections, or other challenges that occur later in postnatal development (P7-P21).

Immune memory is not limited to the adaptive immune system (T and B cells). Innate immune cells, including microglia, also form immune memories that can enhance (prime) or suppress (tolerate responses to a second “hit” [6, 40, 41]. Here we show that LPS-induced inflammation during late postnatal development attenuates expression of some pro-inflammatory genes in response to a second “hit” of LPS as an adult. Of particular interest, we found sex- and region-dependent vulnerability to an early life inflammatory (LPS) challenge. In males, cortical versus spinal microglia were more affected, regardless of postnatal timing. In contrast, spinal microglia were more affected in females vs. males, but only with P7 or P18 LPS challenge. It is not clear why the spinal cord appears to less affected, but we hypothesize distinct cortex vs. spinal cord microenvironments underlie differential microglial reactivity to LPS challenge.

In healthy adult rats, cortex and cervical spinal cord microglia exhibit multiple differences, including differential sensitivities to systemic inflammation. Microglial density is considerably higher in cortex vs. cervical spinal cord [42]. We report here that LPS-evokes more robust upregulation of CXCL10, Ptgs2 and Arg1 in cortical vs. spinal microglia in both males and females. C/EBPb, a transcription factor regulating pro-inflammatory gene expression [43, 44], is also elevated more in cortical vs. spinal microglia in response to LPS. We detected few sex-dependent differences in responses to LPS, although females had weaker IL6 and iNOS responses in cortical microglia, confirming that inflammation in males may be more robust [45].

Microglial activities are shaped by cues from neurons and astrocytes *via* several secreted or membrane-bound factors. Here, we evaluated CSF1 and CX3CL1 (fractalkine), important regulators of microglial survival and activities, respectively [46–48]. At lower concentrations, CX3CL1 has anti-inflammatory properties, although high concentrations are pro-inflammatory [48]. Whereas CX3CL1 expression was unaffected by postnatal LPS, CSF-1 expression was attenuated in both CNS regions and sexes. CSF-1 is necessary for microglial survival and proliferation [49, 50]. Thus, weaker CSF-1 responses in postnatally LPS-challenged rats may limit adult microglial pro-inflammatory responses. CSF1 and CX3CL1 likely contribute to region- and sex-dependent differences in microglial sensitivity to postnatal immune challenge, but other factors such as stress and sex hormones are likely involved. Indeed, estrogens have anti-inflammatory activities and can modify microglial responses to LPS [51, 52].

Studies concerning the effects of early life inflammation have mostly been done in males, with few studies reporting responses from both sexes. Here we show that males and females are affected differently by postnatal inflammation, especially in the spinal cord. In rodents, microglial sex differences emerge as early as E18 [53], with multiple postnatal and adult sex-differences in transcription profile, density and morphology [22, 23, 54, 55]. Some studies report increased numbers of amoeboid microglia in early postnatal males, whereas activated microglia are found in adult females [55, 56]. Because males have a higher proportion of activated microglia during development vs. females, males may be more vulnerable to early life insults [54]. Indeed, we now report microglia are more affected in males than females, particularly in the cortex (not spinal cord). One limitation of the present study is that we did not evaluate the estrous cycle at the time of adult LPS challenge. Since estrogen has anti-inflammatory properties, different estrous cycle phases may obscure differences in LPS responsiveness.

While we did not observe major differences in basal microglial gene expression in adult rats treated with postnatal LPS (cortex or spinal cord), their responses to a second (adult) LPS “hit” were significantly impacted. Other studies reported no impact of neonatal LPS (with the same dose used here) on adult basal spinal inflammatory gene expression or glial morphology [57]. Although we did not evaluate microglial morphology, long-term changes in microglial morphology seem unlikely (but not ruled out) since basal gene expression was unchanged. However, additional studies are needed to determine if and how microglial morphology is affected. Whereas adult microglia, under normal conditions, may appear unaffected by a history of LPS challenge, long-lasting effects of a postnatal inflammatory insult were unmasked by challenging the adult immune system with a “second hit”. Other studies report similar findings; for example, impaired memory after neonatal bacterial infection was revealed only after second immune challenge [38]. Thus, microglial reactivity to adult-onset pathology or trauma may differ between animals with and without a developmental history of inflammation. More studies are needed to fully understand the impact of early life events on adult-onset disease susceptibility, severity or progression.

While the general gene expression pattern in adult cortical microglia after LPS challenge was attenuated in rats with postnatal LPS challenge versus controls (saline during postnatal development), the same pattern was not observed in spinal microglia. In spinal cord, microglial responses were more mixed, with larger variance for at least some genes, and an increased response for certain pro-inflammatory genes. Similar variation between spinal and cortical microglia was not observed in rats that had not received postnatal LPS (Fig. 3). Recent advances in single cell RNA sequencing have revealed microglial heterogeneity across the CNS, and even in sub-fields of the same region [58, 59]. It is plausible that postnatal LPS increased heterogeneity in spinal microglia (e.g., dorsal versus ventral spinal cord), thereby increasing the variability in gene expression of microglia harvested from the entire cervical spinal cord.

Several mechanisms of microglial reprogramming induced by maternal or neonatal inflammation have been suggested, including HPA axis dysfunction and epigenetic modifications [2–6]. In vitro microglial studies demonstrate that aerobic glycolysis induced by LPS leads to innate immune tolerance [60]. We hypothesize that multiple mechanisms contribute to altered adult microglial responses to LPS, including mechanisms within microglia per se as well as inter-cellular or environmental mechanisms. Since both neurons and astrocytes interact with microglia, changes in neuronal or astrocytic functions induced by postnatal inflammation could regulate microglial activities. It is not clear how long the “long-term” impact of postnatal inflammation on microglia persists. Rats exposed to LPS at P7 had a recovery time 11 days longer than rats exposed to LPS at P18 since all adult rats were studied at the same age. Thus, differences in recovery times after postnatal LPS could account for some differences observed in rats treated at different postnatal times.

Because LPS does not cross the blood-brain barrier, neuroinflammation following systemic LPS is largely mediated secondarily, spreading to the CNS *via* molecules upregulated by peripheral inflammation that do cross the blood-brain barrier. Because of this fact, we analyzed select genes in the spleen. Based on these measurements, the adult peripheral immune response did not appear affected by LPS exposure at P7 or P12, whereas responses to LPS at P18 were mixed. Thus, we cannot completely exclude the possibility that long-lasting effects on microglia were at least partially mediated through altered peripheral immune responses.

## Conclusions

Prenatal immune challenges lead to suppressed microglial pro-inflammatory responses in adulthood [61, 62]. Here we show that acute peripheral (and neuro) inflammation induced by LPS during later postnatal development (P7-P18) affected microglial responses to LPS in adult rats. These effects were gene specific and depended on sex, CNS region and the timing of postnatal LPS challenge. Multiple outstanding questions require future research to determine: (1) mechanisms responsible for regional and sex differences in postnatal inflammation effects; (2) the persistence of these changes; (3) whether postnatal inflammation alters responses to other second challenges (e.g., chronic intermittent hypoxia); and (4) consequences of altered adult microglial responses for adult-onset neurodegenerative disorders or acquired neural injuries.

## Electronic supplementary material

Below is the link to the electronic supplementary material.

Supplementary Material 1

Supplementary Material 2

Supplementary Material 3

Supplementary Material 4

## Data Availability

All data supporting the findings of this study are available within the paper. PCR primer information is available in Table 2.
