# Supplementary figures and images for: Acute postnatal inflammation alters adult microglial responses to LPS that are sex-, region- and timing of postnatal inflammation-dependent
Source: J Neuroinflammation. 2024 Oct 10;21:256. doi: 10.1186/s12974-024-03245-x (PMC11465935; doi:10.1186/s12974-024-03245-x)

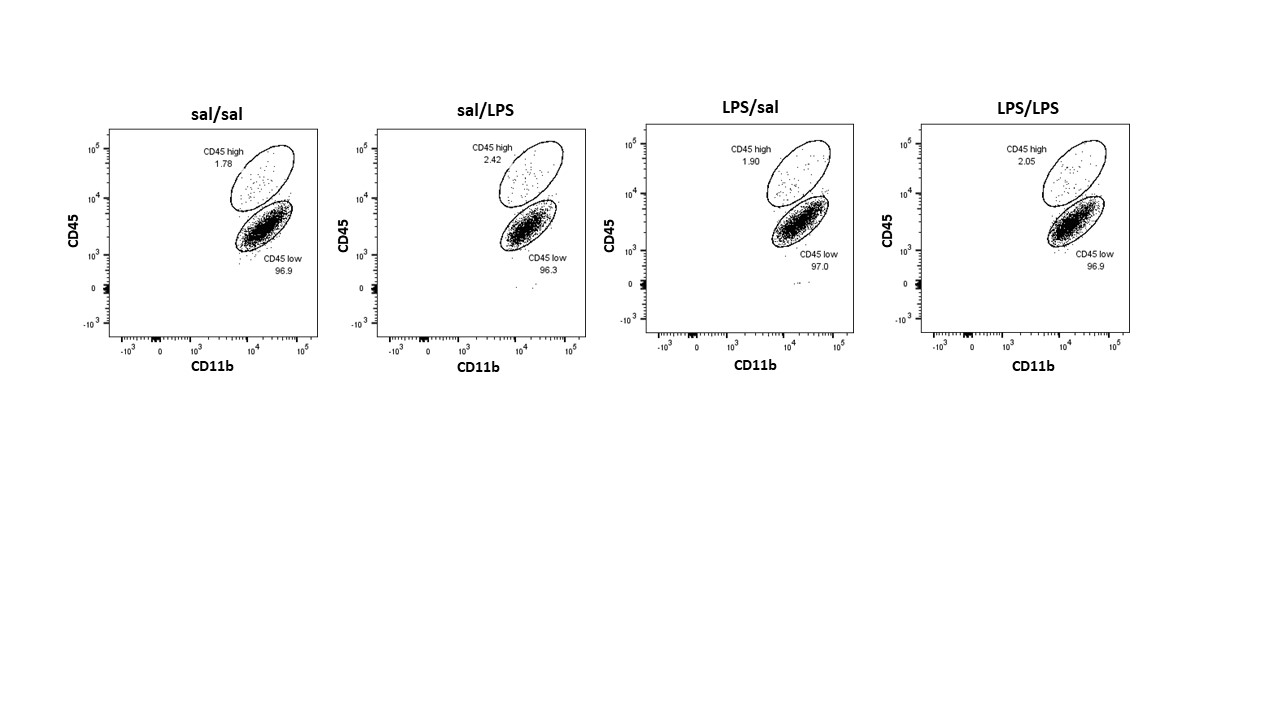

Supplement: Supplementary file 1 — Supplementary Material 1 [file 12974_2024_3245_MOESM1_ESM.jpg]

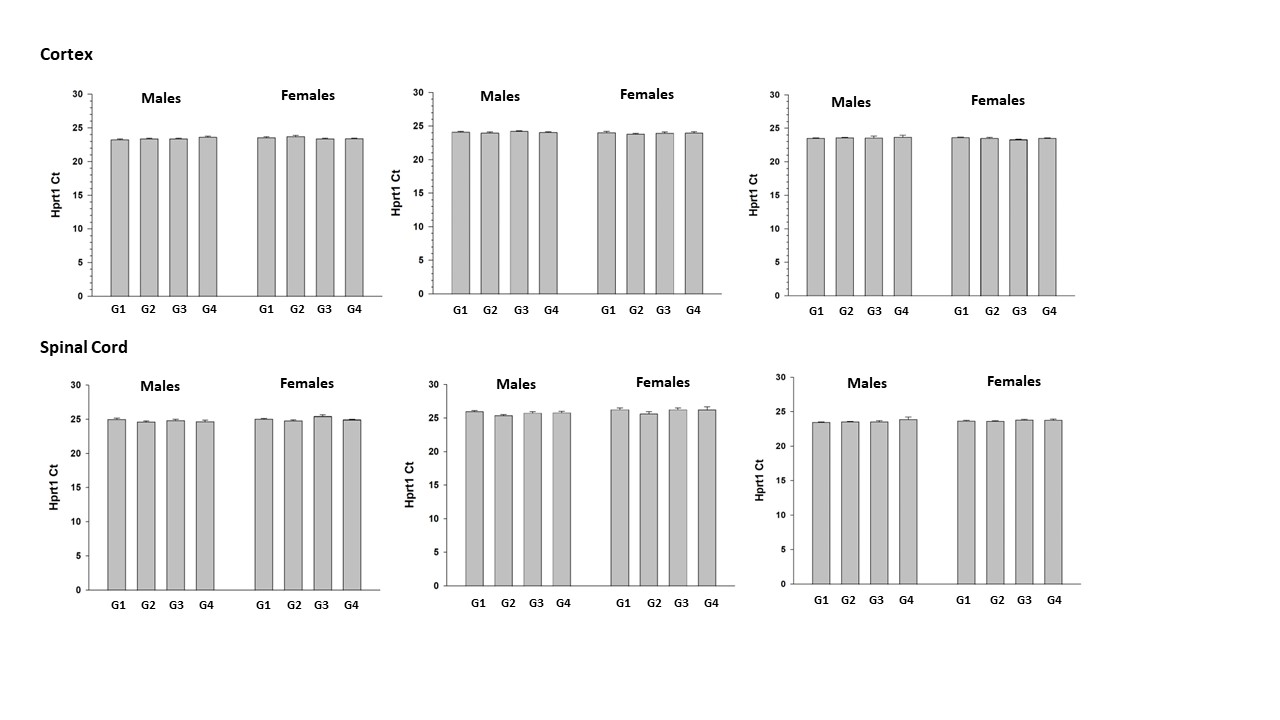

Supplement: Supplementary file 2 — Supplementary Material 2 [file 12974_2024_3245_MOESM2_ESM.jpg]

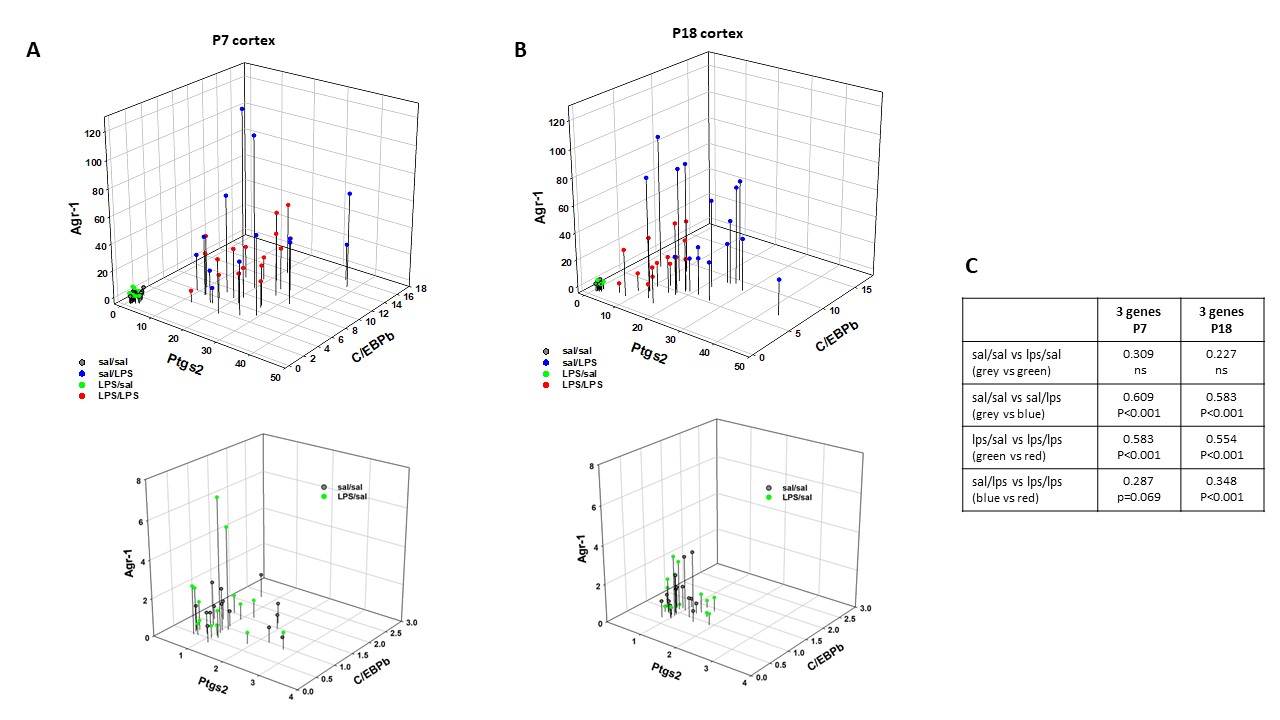

Supplement: Supplementary file 3 — Supplementary Material 3 [file 12974_2024_3245_MOESM3_ESM.jpg]
