# Supplementary material for: Acute postnatal inflammation alters adult microglial responses to LPS that are sex-, region- and timing of postnatal inflammation-dependent
Source: J Neuroinflammation. 2024 Oct 10;21:256. doi: 10.1186/s12974-024-03245-x (PMC11465935; doi:10.1186/s12974-024-03245-x)
Supplement: Supplementary file 4 — Supplementary Material 4 [file 12974_2024_3245_MOESM4_ESM.docx]

**Supplementary information**

**Bray-Curtis and PERMANOVA analysis**

The BC dissimilarity index was calculated as follows:

*BCij* $=1- \frac{2Cij}{Si+Sj}$

Where i,j are experimental groups; Cij is the sum of the lesser count of each species (mRNA); Si and Sj are the total number of specimens in the group. The mRNA fold change was used as the “RNA count”. To calculate fold change, the ΔΔCt of the control group was set to 1 and the experimental group fold change was calculated relative to the control. Thus, for the Bray-Curtis distance calculation, if the fold change was 8.8, the mRNA count was considered to be 8.8 and the control mRNA count was considered to be 1.

We first performed sensitivity analyses to determine if this statistic was suitable to reflect changes between microglial gene expression among groups. As an example, we performed a sensitivity analysis using the expression of 3 genes (C/EBPβ, Ptgs2 and Arg-1), comparing cortical microglia from rats receiving LPS at P7 or P18 versus control rats receiving saline at these ages (Supplementary Fig 3). As illustrated in the 3D graphs in Suppl Figures 3A, B, basal gene expression was comparable, regardless of the postnatal exposure (sal/sal or LPS/sal groups), adult LPS challenge upregulated all 3 genes in the sal/LPS and LPS/LPS groups. Whereas LPS at P7 had little effect on LPS-induced gene responses in adults, P18 LPS challenge attenuated adult microglial responses. The figure illustrates that gene expression in the sal/LPS and LPS/LPS treatment groups were more similar when the postnatal LPS challenge occurred at P7, and they were more distinct when the LPS challenge occurred at P18, indicating greater dissimilarity. The Bray-Curtis dissimilarity indices (Suppl Figure 3C) comparing basal gene expression in control animals (sal/sal) versus rats exposed to LPS at P7 or P18 (LPS/sal groups) were 0.309 and 0.227, indicating high similarity between these groups. In contrast, the dissimilarity indices were high (and statistically significant) comparing animals receiving adult LPS treatment vs saline treatment: 0.609 and 0.583 for rats that had received saline at P7 or P18 (sal/sal vs sal/LPS); and 0.583 and 0.554 for animals receiving LPS at P7 or P18 (LPS/sal vs LPS/LPS). We found statistically significant differences in the adult LPS response in rats receiving LPS also at P18 (BC index: 0.348), but not at P7 (BC Index 0.287). In Suppl Fig 3A and B we see that samples from sal/LPS and LPS/LPS groups (blue vs red) clustered closer together in the P7 samples than did the P18 cortex samples, indicating that P7 cortex samples were more “similar” than samples from the P18 cortex. Accordingly, samples from the P18 cortex had a higher dissimilarity index (0.348) than those from P7 cortex (0.287).

**Supplementary Figure legends**

**Suppl Figure 1: Analysis of CD11b cells by flow cytometry.** Cortex tissues were dissociated into single cell suspensions and stained with fluorochrome-conjugated CD11b (PE) and CD45 (APC) antibodies. Samples were then analyzed by flow cytometry. Samples in the figure are from adult animals treated at P18 with saline or LPS. As adults, the rats were treated with saline or LPS. LPS did not increase the CD11b^+^ CD45^high^ population (presumed macrophages). Similar results were obtained from the spinal cord and from animals receiving LPS treatment at P7 or P12.

**Suppl Figure 2: Ct values for the Hprt1 gene.** The expression of the housekeeping gene Hprt1 was similar between groups (G1-G4); LPS treatment did not affect its expression. G1: sal/sal; G2: sal/LPS; G3: LPS/sal; G4: LPS/LPS

**Suppl Figure 3: Bray-Curtis analysis of differences in microglial responsiveness to LPS.** Illustration of C/EBPb, Arg-1 and Ptgs2 mRNA expression in cortical microglia. To visualize the impact of postnatal LPS challenge on adult microglial responses to a second LPS challenge, the expression of the 3 most affected genes was compared in microglia exposed to LPS or saline at P7 **(A)** and P18 **(B)**. The basal expression of these genes was comparable in adult animals receiving saline (grey) or LPS (green) during postnatal development. The bottom figures show basal gene expression on a different scale. LPS at 12 weeks of age increased expression of all three genes (red, blue), however, we did not see a clear separation between animals receiving saline (blue) or LPS (red) at P7. On the other hand, there was distinct grouping when the postnatal treatment was received at P18, suggesting differential responses of microglia with and without prior LPS exposure. Data from both sexes are pooled. **(C)** Sensitivity analysis of quantifying group differences by Bray-Curtis dissimilarity index. This index measures the distance between individual points within and between groups. As expected, the dissimilarity index was highest when basal and LPS induced gene expression were compared. On the other hand, the index was lower (suggesting greater similarity) for basal gene expression in animals that received either saline or LPS postnatally. Statistical significance was determined by a PERMANOVA test. ns, not significant.
